# Significant Stability Enhancement in High‐Efficiency Polymer:Fullerene Bulk Heterojunction Solar Cells by Blocking Ultraviolet Photons from Solar Light

**DOI:** 10.1002/advs.201500269

**Published:** 2015-11-17

**Authors:** Jaehoon Jeong, Jooyeok Seo, Sungho Nam, Hyemi Han, Hwajeong Kim, Thomas D. Anthopoulos, Donal D. C. Bradley, Youngkyoo Kim

**Affiliations:** ^1^ Organic Nanoelectronics LaboratorySchool of Applied Chemical EngineeringKyungpook National UniversityDaegu702‐701Republic of Korea; ^2^ Center for Plastic ElectronicsDepartment of PhysicsBlackett LaboratoryImperial College LondonLondonSW7 2AZUK; ^3^ Priority Research CenterResearch Institute of Advanced Energy TechnologyKyungpook National UniversityDaegu702‐701Republic of Korea; ^4^ Division of Mathematical, Physical and Life SciencesUniversity of OxfordOxfordOX1 3PDUK

**Keywords:** degradation, morphology, polymer:fullerene solar cells, stability, UV light

## Abstract

Achievement of extremely high stability for inverted‐type polymer:fullerene solar cells is reported, which have bulk heterojunction (BHJ) layers consisting of poly[4,8‐bis(5‐(2‐ethylhexyl)thiophen‐2‐yl)benzo[1,2‐b:4,5‐b′]dithiophene‐alt‐3‐fluorothieno[3,4‐b]thiophene‐2‐carboxylate] (PTB7‐Th) and [6,6]‐phenyl‐C71‐butyric acid methyl ester (PC_71_BM), by employing UV‐cut filter (UCF) that is mounted on the front of glass substrates. The UCF can block most of UV photons below 403 nm at the expense of ≈20% reduction in the total intensity of solar light. Results show that the PTB7‐Th:PC_71_BM solar cell with UCF exhibits extremely slow decay in power conversion efficiency (PCE) but a rapidly decayed PCE is measured for the device without UCF. The poor device stability without UCF is ascribed to the oxidative degradation of constituent materials in the BHJ layers, which give rise to the formation of PC_71_BM aggregates, as measured with high resolution and scanning transmission electron microscopy and X‐ray photoelectron spectroscopy. The device stability cannot be improved by simply inserting poly(ethylene imine) (PEI) interfacial layer without UCF, whereas the lifetime of the PEI‐inserted PTB7‐Th:PC_71_BM solar cells is significantly enhanced when UCF is attached.

## 1 Introduction

Great attention has been paid to organic solar cells for the last two decades because of their advantages, including easy tailoring of solar light absorption with various organic materials and low temperature processes leading to inexpensive solar cells, over conventional inorganic solar cells.^1^, ^2^, ^3^, ^4^, ^5^, ^6^ The power conversion efficiency (PCE) of organic solar cells has been noticeably improved up to 8%–10% for single‐stack devices and 11.5% for tandem devices,^7^, ^8^, ^9^, ^10^, ^11^, ^12^, ^13^, ^14^, ^15^, ^16^ since early works for the bulk heterojunction (BHJ) concept and the BHJ nanomorphology control.^17^, ^18^, ^19^, ^20^, ^21^, ^22^, ^23^, ^24^, ^25^ Interestingly, most of the high‐efficiency (>8%) organic solar cells are fabricated with blends of conjugated polymers and soluble fullerenes, the so‐called polymer:fullerene solar cells, because fullerene derivatives possess desirable energy band structures and high electron mobilities.^26^, ^27^, ^28^

Although the efficiency of polymer:fullerene solar cells has been considerably improved as mentioned above, their stability (or lifetime) is still too poor to consider commercialization.^29^, ^30^ Of various factors affecting to the stability of polymer:fullerene solar cells, the acidity issue of hole‐collecting buffer layers, typically poly(3,4‐ethylenedioxythiophene):poly(styrenesulfonate) (PEDOT:PSS)) that is broadly used for normal‐type devices with indium‐tin oxide (ITO) electrodes, has been suggested to be overcome by controlling the acidity with addition of base materials.^31^, ^32^ The degradation of metal electrodes can be easily resolved by applying hermetic encapsulations.^33^, ^34^, ^35^ However, in the case of high‐efficiency polymer:fullerene solar cells, no clear methods have been so far reported for overcoming the degradation of polymer:fullerene BHJ layers that have electron‐donating conjugated polymers with benzodithiophene (BDT) units in the main chains such as poly[4,8‐bis[(2‐ethylhexyl)oxy]benzo[1,2‐b:4,5‐bi′]dithiophene‐2,6‐diyl][3‐fluoro‐2‐[(2‐ethylhexyl)carbonyl]thieno[3,4‐b]‐thiophenediyl] (PTB7), poly[4,8‐bis(5‐(2‐ethylhexyl)thiophen‐2‐yl)benzo[1,2‐b:4,5‐b′]dithiophene‐alt‐3‐fluorothieno[3,4‐b]thiophene‐2‐carboxylate] (PTB7‐Th), etc.^36^, ^37^ We note that the device stability could be slightly improved by optimization of thermal annealing process in the case of low efficiency (<5%) devices with BHJ layers of poly(3‐hexylthiophene) (P3HT) and [6,6]‐phenyl‐C61‐butyric acid methyl ester (PC_61_BM). However, on‐going aggregation of fullerene derivatives in the BHJ layers has been shown to be a major reason for the poor longtime stability of polymer:fullerene solar cells.^38^, ^39^

In terms of PTB7 derivatives, the conjugated heterocyclic groups including BDT and thienothiophene (TTP) units in the polymer main chain are considered to be quite vulnerable to photodegradation under illumination with solar radiation.^40^, ^41^ As most conjugated organic compounds have double bonds (pi‐orbitals), their photodegradation undergoes by cleavage of double bonds under UV lights.^42^, ^43^ The BDT and TTP units have pi‐orbitals in a fused heterocyclic structure so that their double bonds can be strongly influenced by the UV light that is about 5% of total solar flux.^44^ In addition, the morphology of polymer:fullerene BHJ layers may be affected by the degradation of the BDT and TTP units under sun light because the initial conformation of polymer chains can be deformed by disruption of conjugated bond structures in the polymer main chains.

In this work, we have attempted to block the UV light from the simulated solar radiation by employing a UV‐cut filter (UCF) (cutoff = 405 nm) and then examined the stability of polymer:fullerene solar cells with BHJ layers of PTB7‐Th and [6,6]‐phenyl‐C71‐butyric acid methyl ester (PC_71_BM). In order to rule out the acidity effect of PEDOT:PSS in the case of normal‐type devices, inverted‐type devices were fabricated by introducing zinc oxide (ZnO) as an electron‐collecting buffer layer and molybdenum oxide (MoO_3_) as a hole‐collecting buffer layer. To understand the trend of stability with exposure time under illumination with solar light, the nanomorphology and atom composition of BHJ layers was measured with transmission electron microscopy (TEM). Furthermore, the possible change of atom environments in the BHJ layers was investigated with X‐ray photoelectron spectroscopy (XPS). Finally, the PTB7‐Th:PC_71_BM solar cells with poly(ethylene imine) (PEI) interfacial dipole layers were fabricated and subjected to the stability test.

## 2 Results and Discussion

**Figure 1:**
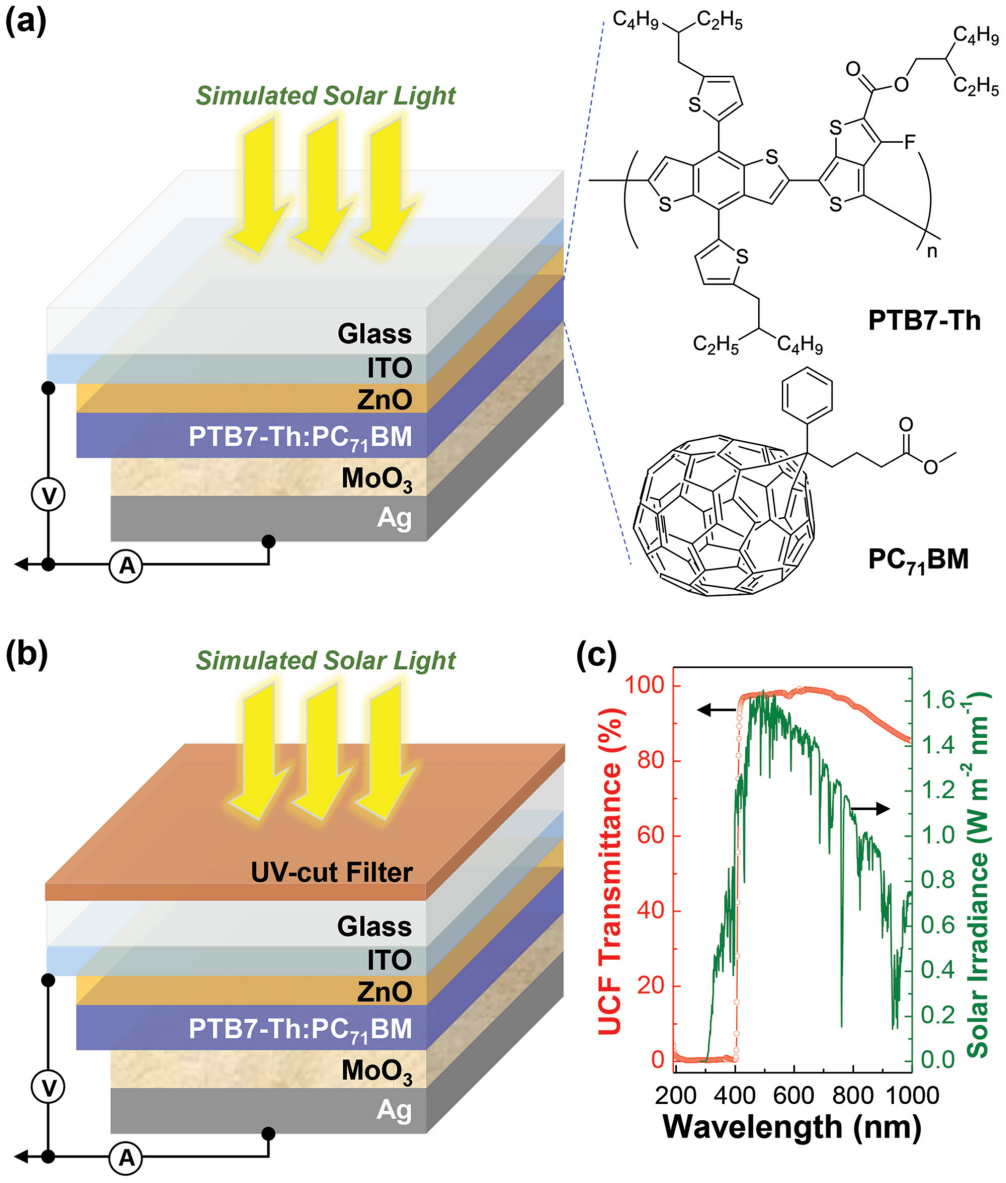
Structure of glass/ITO/ZnO/PTB7‐Th:PC_71_BM/MoO_3_/Ag solar cells without a) and with b) UCF (see the chemical structure of PTB7‐Th and PC_71_BM in panel (a)). c) Optical transmittance of UCF (left) and solar irradiance (right).

As shown in **Figure** **1**a, inverted‐type solar cells (glass/ITO/ZnO/PTB7‐Th:PC_71_BM/MoO_3_/Ag) were fabricated by placing the BHJ (PTB7‐Th:PC_71_BM = 1:1.5 by weight) layer in between the ZnO layer (electron‐collecting buffer role) and the MoO_3_ layer (hole‐collecting buffer role). To examine the influence of UV light, a UCF was attached to the front of glass substrate as illustrated in Figure 1b. Here we note that the present UV‐cut filter blocks most of UV photons below 405 nm (wavelength) out of the solar spectrum so that the overall intensity of incident solar radiation can be decreased by ≈5% in the presence of additional intensity reduction at the wavelengths of >700 nm (see Figure 1c). The final intensity by the presence of UCF was measured ≈80% of the initial intensity of the simulated solar light (note that the intensity measurement was carried out with a calibrated photodiode so that a spectral mismatch was not considered here—see the Experimental Section). Thus the initial efficiency is expected to be lower by ≈20% for devices with UCF than those without UCF.

**Figure 2:**
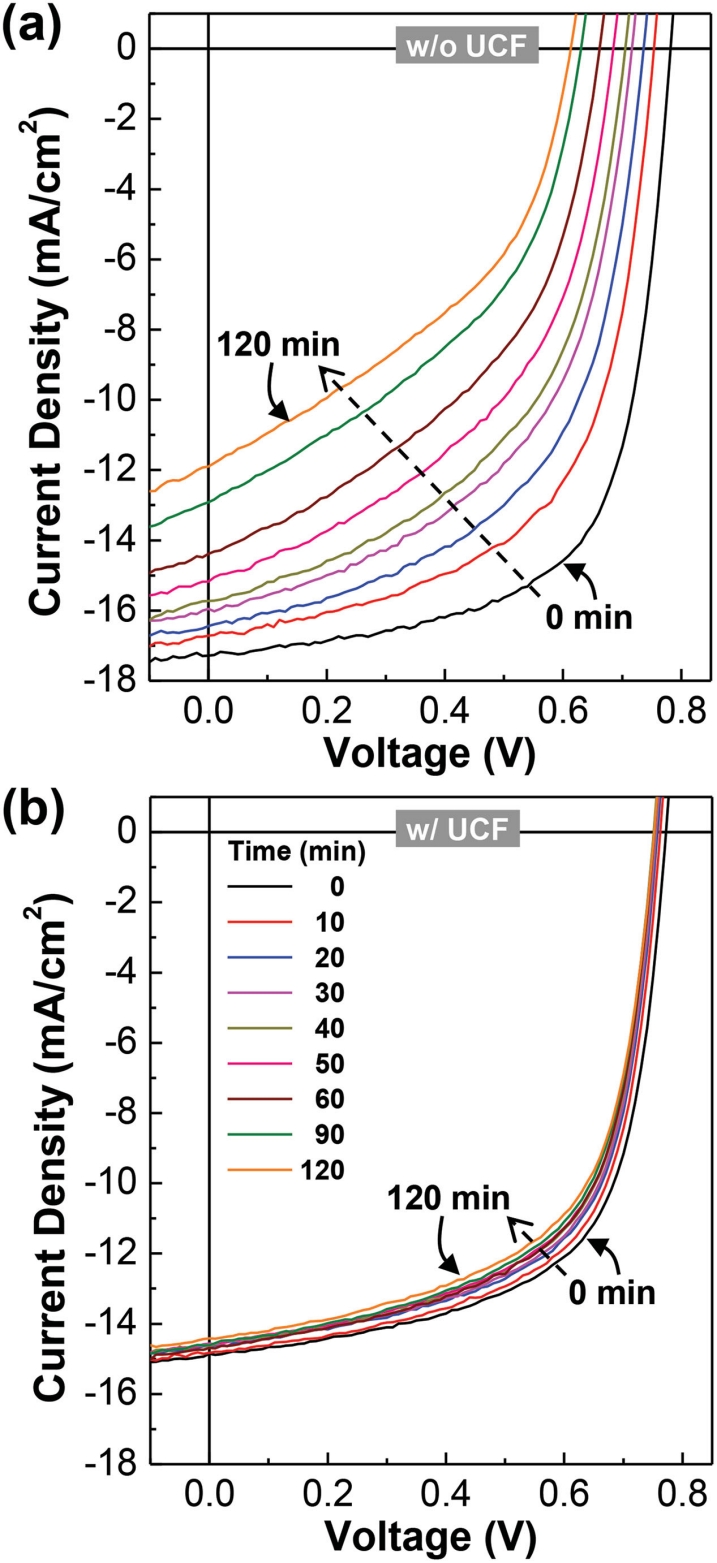
Current density–voltage (*J–V*) curves of glass/ITO/ZnO/PTB7‐Th:PC_71_BM/MoO_3_/Ag solar cells under continuous illumination with simulated solar light (air mass 1.5G, 100 mW cm^−2^) for 120 min: a) without UCF and b) with UCF. Note that the incident light intensity was reduced by ≈20% for the device with UCF.

The stability of devices fabricated as described in Figure 1 was tested by measuring current density–voltage (*J–V*) curves under illumination with solar light (air mass 1.5G, 100 mW cm^−2^). As shown in **Figure** **2**a, the *J–V* curve of the device without UCF became noticeably poor under continuous illumination with the simulated solar light. Both short circuit current density (*J*_SC_) and open circuit voltage (*V*_OC_) were remarkably reduced under solar light illumination for 120 min. However, surprisingly, very little change in the *J–V* curves was measured for the device with UCF even after 120 min under solar light illumination. To examine whether the reduced (≈20%) light intensity influenced the slow change of *J–V* curves for the device with UCF, the device without UCF was subject to the stability test under illumination with the solar light with the reduced intensity (air mass 1.5G, 80 mW cm^−2^). As shown in Figure S1, the change of *J–V* curves was still larger for the device without UCF under illumination with 80 mW cm^−2^ solar light than that with UCF under illumination with 100 mW cm^−2^ solar light, even though the extent of change for the device without UCF was relatively smaller under 0.8 sun (80 mW cm^−2^) than 1 sun (100 mW cm^−2^). This result reflects that the presence of UV light in the incident solar light is mainly responsible for the rapid change of *J–V* curves for the device without UCF even though 20% of the initial intensity was cut out.

**Figure 3:**
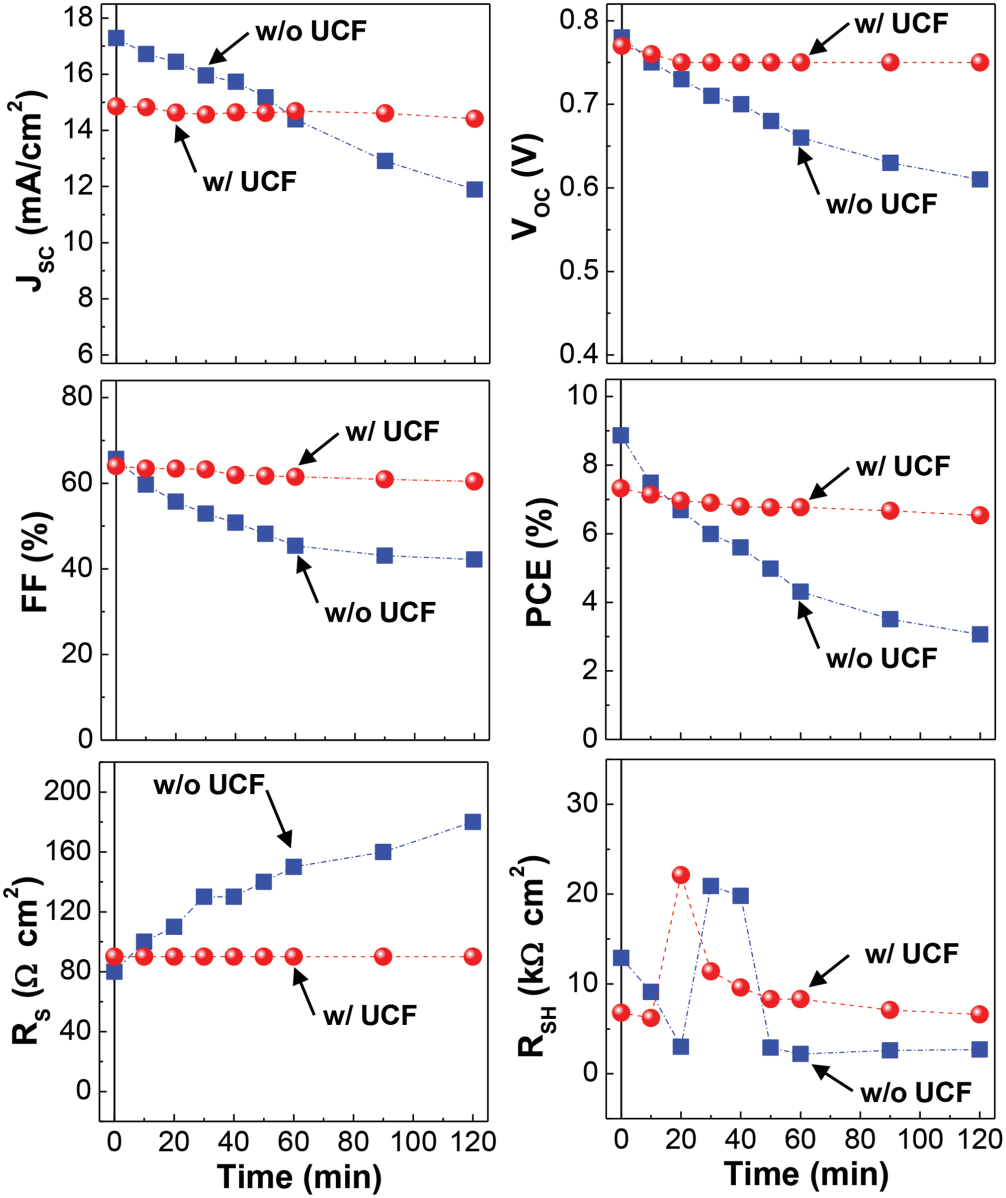
*J*_SC_, *V*_OC_, FF, PCE, *R*_S_, and *R*_SH_ as a function of exposure time under simulated solar light (air mass 1.5G, 100 mW cm^−2^) for glass/ITO/ZnO/PTB7‐Th:PC_71_BM/MoO_3_/Ag solar cells with and without UCF.

To investigate the detailed trends according to the presence of UCF, all solar cell parameters extracted from the *J–V* curves in Figure 2 are plotted as a function of exposure time (see **Figure** **3**). As expected, *J*_SC_ was rapidly decreased with time for the device without UCF (from 17.29 to 11.89 mA cm^−2^ after 120 min), while it was slightly changed for the device with UCF (from 14.85 to 14.41 mA cm^−2^ after 120 min). The similar trend was observed for *V*_OC_ (without UCF: 0.78 to 0.61 V after 120 min; with UCF: 0.77 to 0.75 V after 120 min). Different from the linear *J*_SC_ reduction with time, an exponential‐like decay in fill factor (FF) was measured in the device without UCF, which may be related to the reduced rate of charge transport inside the PTB7‐Th:PC_71_BM layer (see Table S1 and Figure S2 in the Supporting Information). As a consequence, the PCE decay was extremely smaller for the device with UCF than that without UCF. The large PCE decay for the device without UCF can be also supported by the gradual increase in series resistance (*R*_S_) with time, whereas *R*_S_ was almost stabilized after the initial change in the case of the device with UCF. However, no systematic trend was observed for shunt resistance (*R*_SH_) for both devices but the initial change before 60 min can be attributable to the on‐going change of BHJ and/or interface nanomorphology.

**Figure 4:**
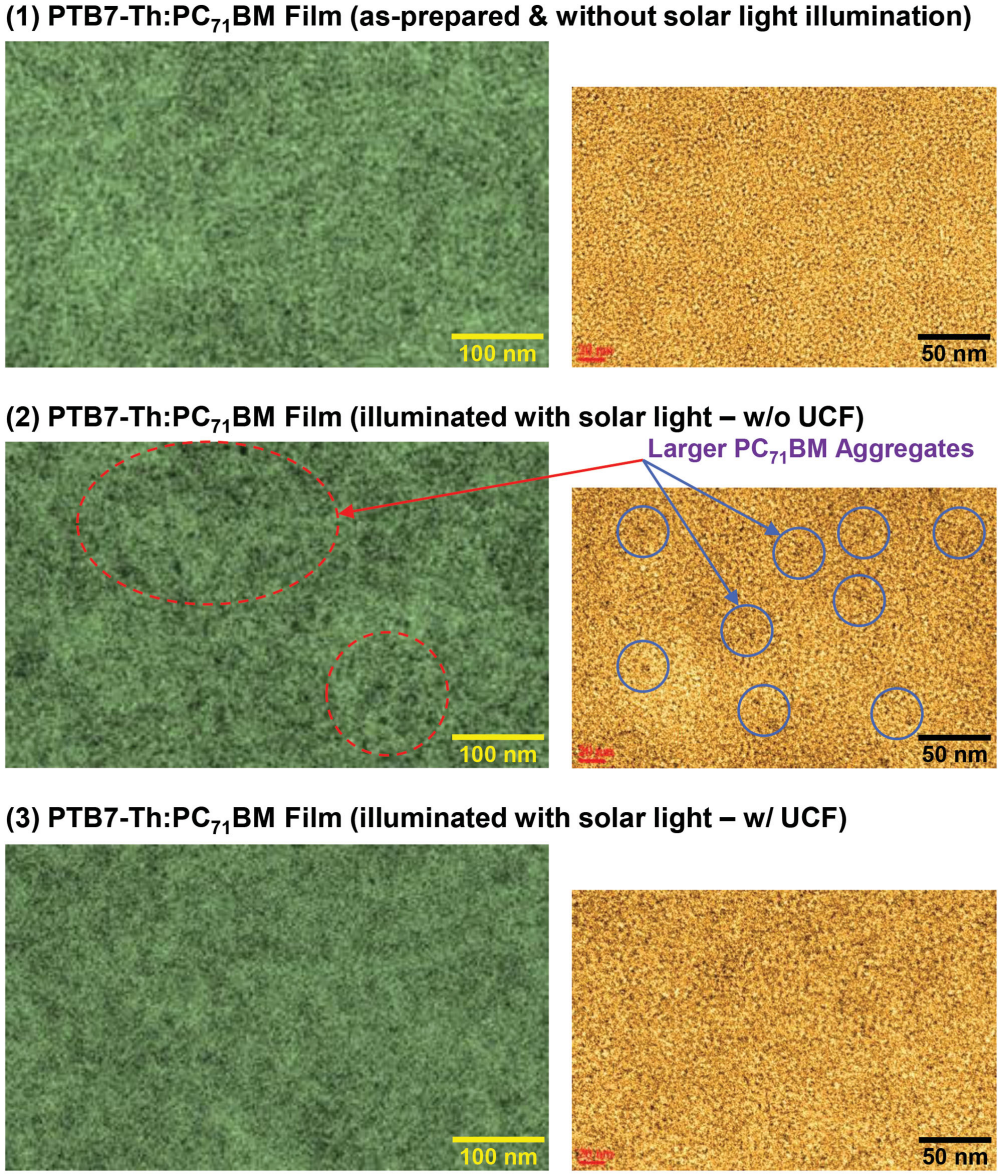
TEM images for PTB7‐Th:PC_71_BM BHJ films: (1) as‐prepared, (2) illuminated with simulated solar light (air mass 1.5G, 100 mW cm^−2^) without UCF for 120 min, and (3) illuminated with simulated solar light (air mass 1.5G, 100 mW cm^−2^) with UCF for 120 min. Note that the right TEM images were separately taken in order to find any more nanomorphology.

To understand the trend of device stability, the PTB7‐Th:PC_71_BM BHJ films were investigated before and after solar light illumination. Optical microscopy measurements showed that all BHJ films were optically clear without any microscale aggregates even after 120 min illumination irrespective of the presence of UCF (see Figure S3 in the Supporting Information). However, interestingly, TEM measurements revealed that apparent nanosized aggregates were formed in the BHJ film illuminated with solar light without UCF, even though such nanosized aggregates were hardly observed for the BHJ film illuminated with solar light with UCF (see **Figure** **4**). The nanoaggregates are considered clusters of PC_71_BM molecules, which might be evolved by the environmental change of components (PTB7‐Th and PC_71_BM) under solar light illumination. Because both PTB7‐Th and PC_71_BM components possess double bonds that are vulnerable to UV photons, the formation of such PC_71_BM aggregates can be attributable to the degradation of both components even though the deterioration of PTB7‐Th polymer is regarded first as studied recently.^16^, ^45^

**Figure 5:**
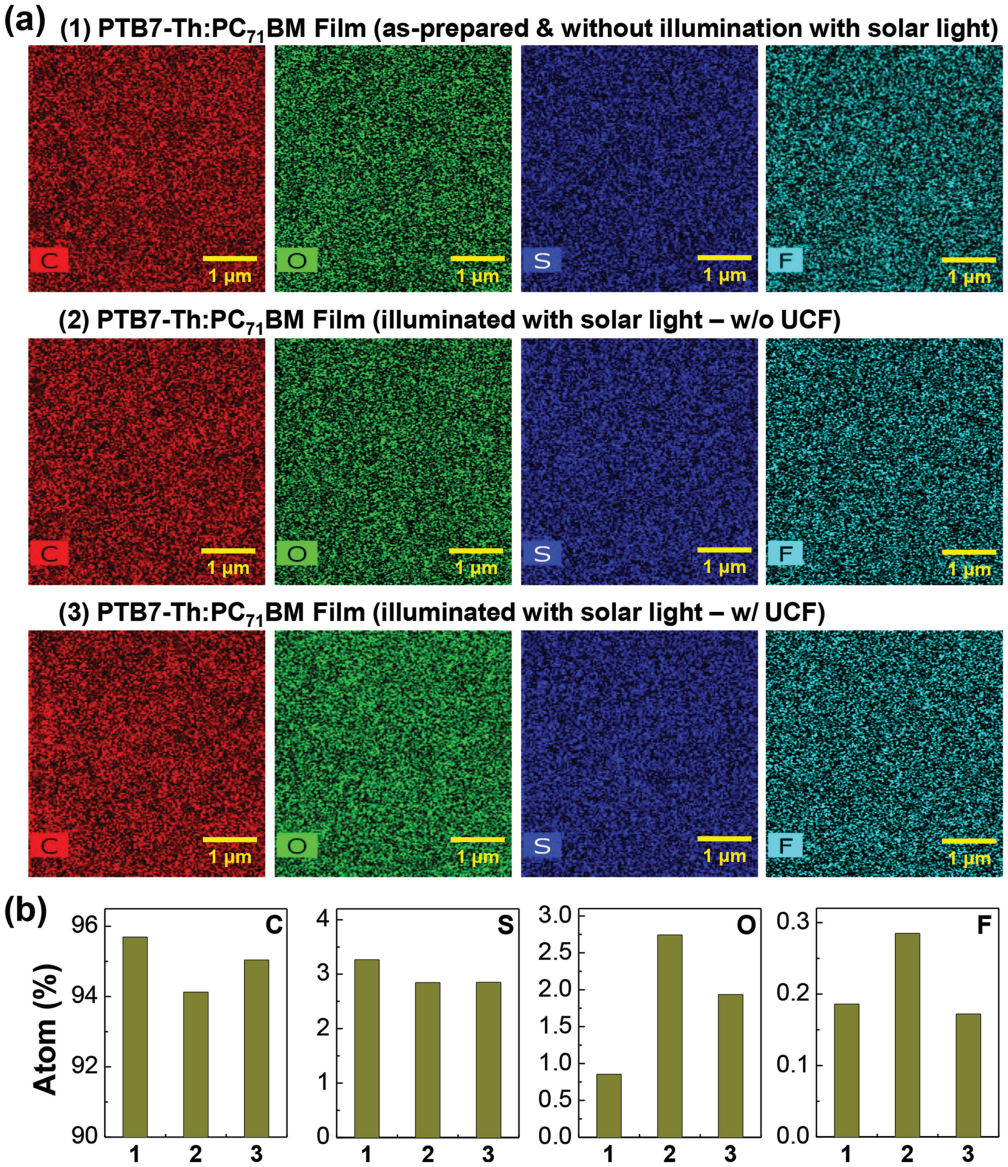
a) STEM images (from left: carbon, oxygen, sulfur, and fluorine) for PTB7‐Th:PC_71_BM BHJ films: (1) as‐prepared, (2) illuminated with simulated solar light (air mass 1.5G, 100 mW cm^−2^) without UCF for 120 min, and (3) illuminated with simulated solar light (air mass 1.5G, 100 mW cm^−2^) with UCF for 120 min. b) Atom compositions (from left: carbon, oxygen, sulfur, and fluorine) obtained by integrating the area (4.3 μm 4.3 μm) of STEM images in panel (a) (note that “1,” “2,” and “3” in the horizontal axis correspond to (1), (2), and (3) in panel (a)).

Next, the PTB7‐Th:PC_71_BM BHJ films was measured using a scanning TEM (STEM) but the atomic distribution on a nanoscale did not deliver any particular distribution (morphology) (see 2D images in **Figure** **5**a). However, as shown in Figure 5b, some changes in atomic compositions were obtained by integration of total area (4.3 μm × 4.3 μm) on each image.^46^, ^47^ Interestingly, the oxygen composition was considerably increased after solar light illumination for 120 min, which was more pronounced for the BHJ film without UCF than that with UCF. This result implies that the PTB7‐Th and/or PC_71_BM components might be oxidized under solar light illumination, which might be caused by the reaction between UV‐activated double bonds and oxygen molecules included in the course of solution preparation and coating processes. In particular, it is worthy to note that the composition of carbon atoms was less changed for the BHJ film with UCF than that without UCF, which may partly explain the different performance decay with exposure time (see Figure 3).

**Figure 6:**
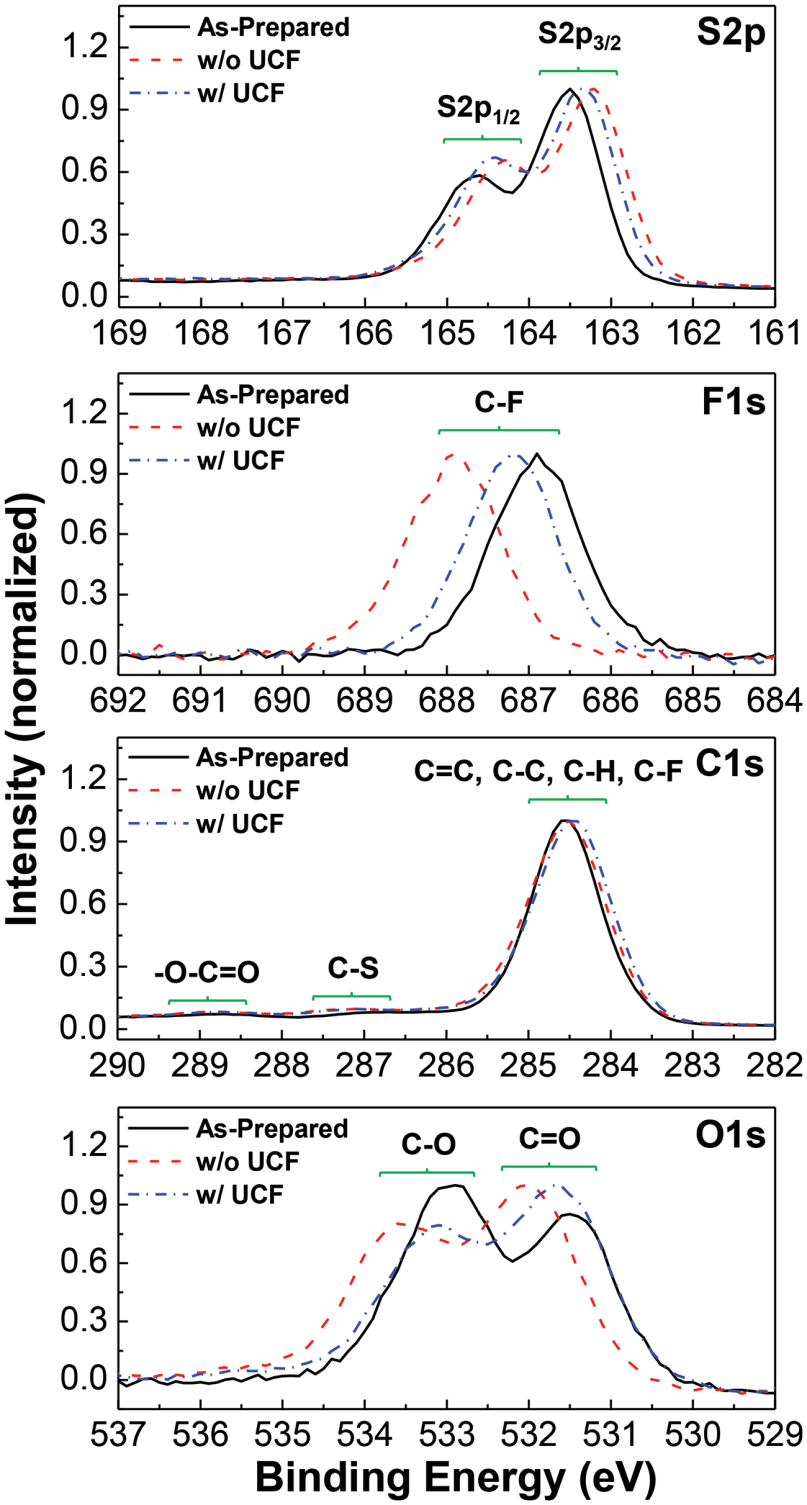
XPS spectra for PTB7‐Th:PC_71_BM BHJ films: (1) as‐prepared (black solid line), (2) illuminated with simulated solar light (air mass 1.5G, 100 mW cm^−2^) without UCF for 120 min (red dashed line), and (3) illuminated with simulated solar light (air mass 1.5G, 100 mW cm^−2^) with UCF for 120 min (blue dashed‐dotted line).

In order to further understand the nanomorphology and atom composition changes measured by TEM and STEM, the environment of atoms in the BHJ films was studied with XPS. As shown in **Figure** **6**, the S2p peak was shifted toward lower energy region after solar light illumination for 120 min irrespective of using UCF, but the peak shift was more pronounced for the BHJ film without UCF. In contrast, the F1s peak was shifted toward higher energy region after solar light illumination for 120 min, and similarly the larger peak shift was made for the BHJ film without UCF (note that the relatively large shifts in F1s peaks compared to other atoms can be partly attributable to the large environmental change of fluorine atoms which might be caused by the degradation of fluorothienothiophene units in PTB7‐Th). Both sulfur and fluorine atoms are included only in PTB7‐Th (not in PC_71_BM) so that the shift of both S2p and F1s peaks may reflect the change of atom environments in PTB7‐Th by solar light illumination, which supports the oxidation of PTB7‐Th as discussed in Figure 5. The oxidized states in the BHJ films are also evidenced from the change of O1s peaks (see bottom panel in Figure 6) in which the intensity ratio of C=O peaks to C—O peaks was inverted after solar light illumination. In addition, the shift of O1s peaks was more pronounced for the BHJ film without UCF than that with UCF. Similarly, the shift of C1s peaks for —O—C=O and C—S groups was relatively larger for the BHJ film without UCF than that with UCF, even though these peaks are considerably lower than other C1s peaks due to their lower concentrations in the materials (see the enlarged spectrum in Figure S7, Supporting Information).

**Figure 7:**
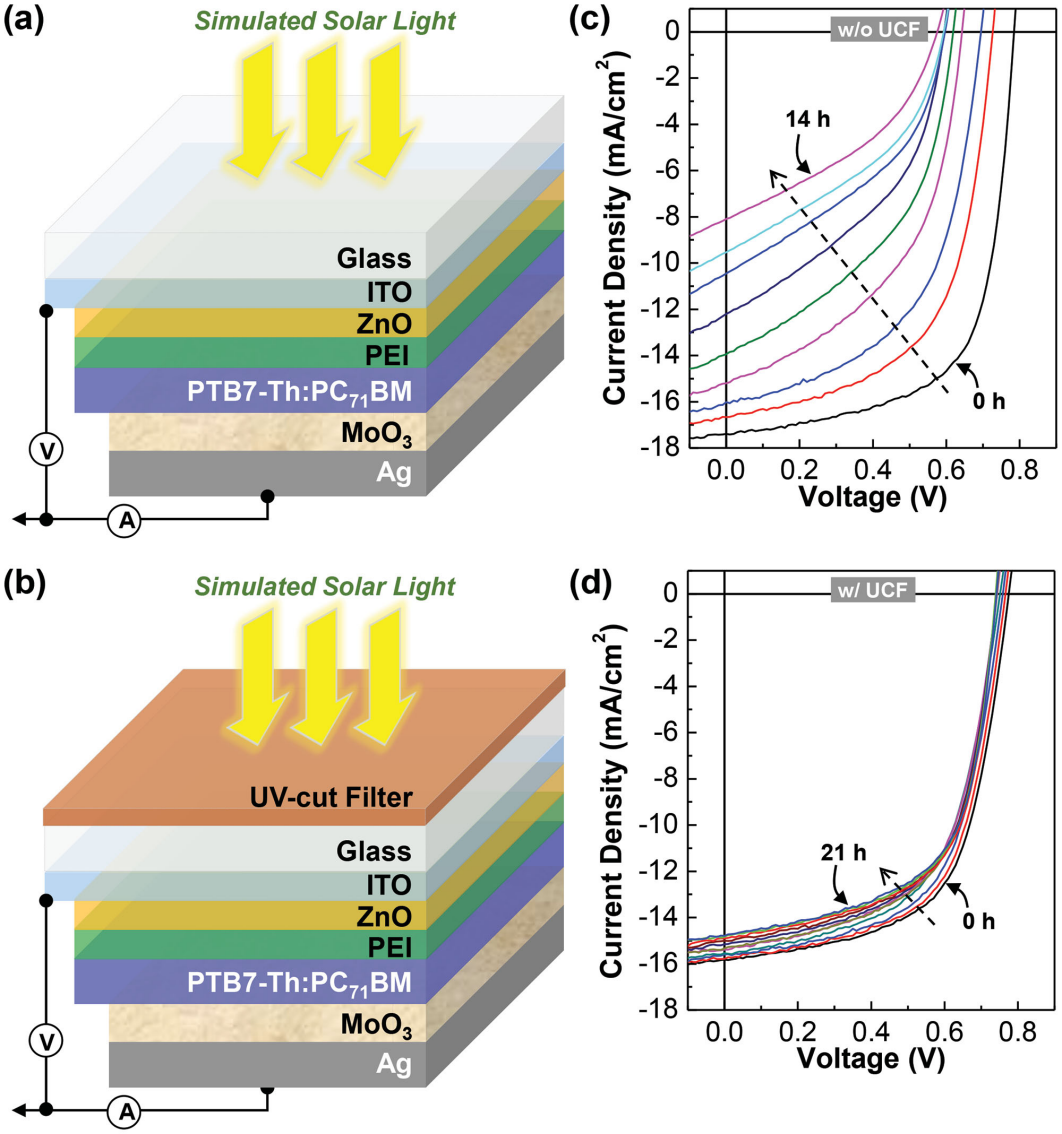
a,b) Structure of glass/ITO/ZnO/PEI/PTB7‐Th:PC_71_BM/MoO_3_/Ag solar cells without a) and with b) UCF. c,d) Current density–voltage (*J–V*) curves of glass/ITO/ZnO/PTB7‐Th:PC_71_BM/MoO_3_/Ag solar cells under continuous illumination with simulated solar light (air mass 1.5G, 100 mW cm^−2^): c) without UCF for 14 h and d) with UCF for 21 h. Note that the incident light intensity was reduced by ≈20% for the device with UCF

**Figure 8:**
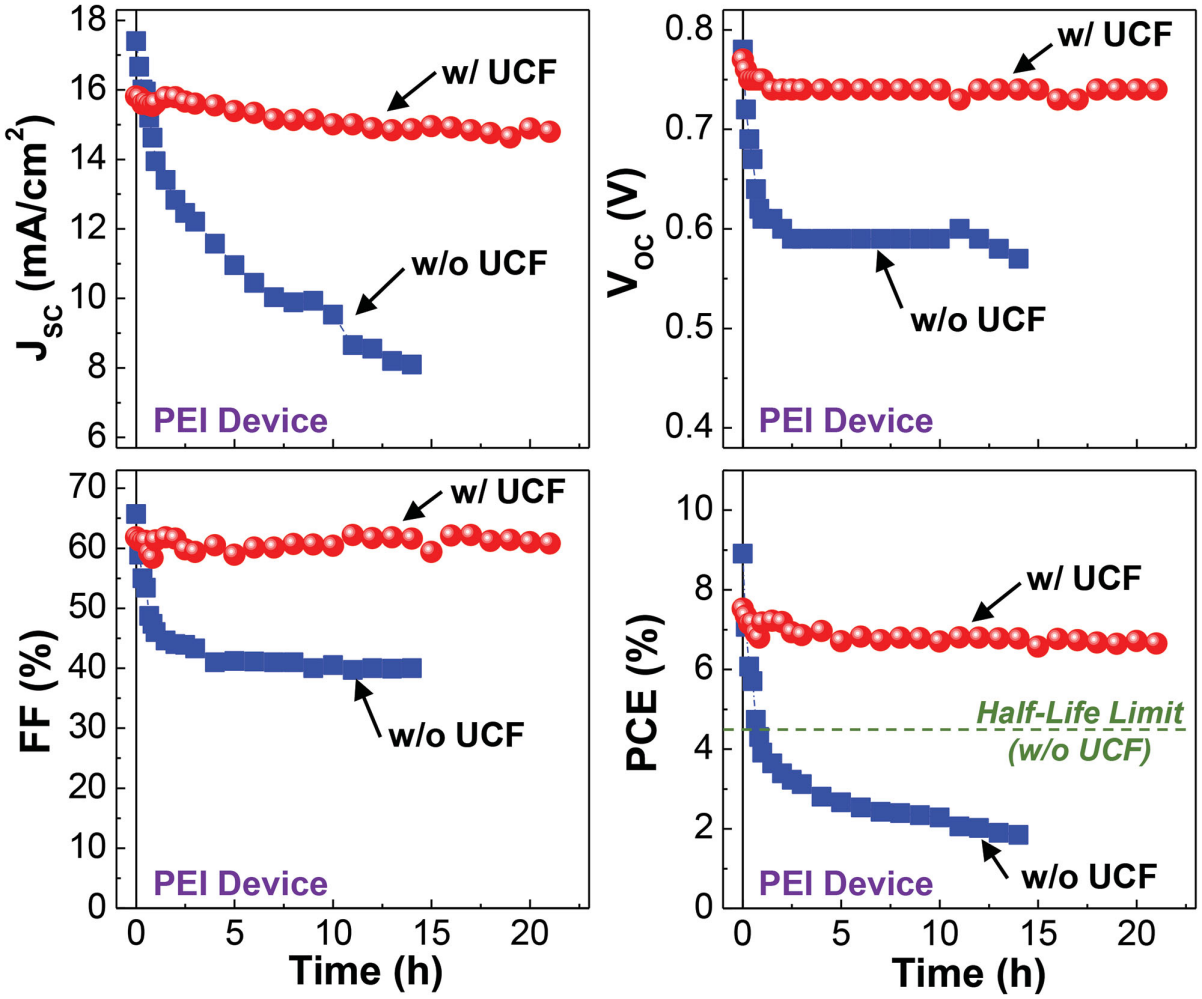
*J*_SC_, *V*_OC_, FF, and PCE as a function of exposure time under a simulated solar light (air mass 1.5G, 100 mW cm^−2^) for glass/ITO/ZnO/PEI/PTB7‐Th:PC_71_BM/MoO_3_/Ag solar cells with and without UCF. Note that the initial PCE was ≈9% for the device without UCF, while it was decreased by ≈20% for the device with UCF.

Finally, the inverted‐type PTB7‐Th:PC_71_BM solar cells with the PEI interfacial layers were fabricated and subjected to the stability test with and without UCF (see **Figure** **7**a,b). As shown in Figure 7c, the device without UCF exhibited gradually poor *J–V* curves with time under continuous illumination with solar light (see full *J–V* curves in Figure S8 in the Supporting Information). However, when UCF was attached, the *J–V* curves were very little changed even after 21 h illumination with solar light. This result indicates that the presence of PEI is not quite helpful for the device stability but UCF is of crucial importance in keeping the device stability. The detailed trends of solar cell parameters are plotted as a function of exposure time in **Figure** **8**a. *J*_SC_ and FF were rapidly decreased for the device without UCF (see the increasing trend of *R*_S_ in Table S2 in the Supporting Information), whereas the decay of *J*_SC_ and FF was very small for the device with UCF. *V*_OC_ was similarly decreased with time for the device without UCF but it was well maintained after marginal drop for the device with UCF. This result reflects that the presence of PEI was not quite beneficial but UCF played an important role in enhancing the device stability.

## 3 Conclusion

The stability of PTB7‐Th:PC_71_BM solar cells with and without UCF was investigated under continuous illumination with a simulated solar light. The performance of solar cells was rapidly decayed for the device without UCF, whereas the device with UCF exhibited extremely slow decay. The low stability of the device without UCF was attributed to the formation of PC_71_BM aggregates in the PTB7‐Th:PC_71_BM BHJ layers, which might be accelerated by the UV‐induced oxidative degradation of PTB7‐Th (mostly), as evidenced by STEM and XPS measurements. The gradually increased *R*_S_ of the device without UCF under illumination with solar light supports the oxidative degradation in the BHJ layers because the semiconducting property of components in the BHJ layers was gradually lost owing to the disruption of conjugations (double bonds) by UV light. The similar poor stability was measured for the PTB7‐Th:PC_71_BM solar cells with the PEI interfacial layers without UCF. Hence the present study suggests that the stability of high‐efficiency polymer:fullerene solar cells are significantly improved if UV light could be effectively blocked.

## 4 Experimental Section

*Materials and Solutions*: PTB7‐Th (weight‐average molecular weight = 126 kDa, polydispersity index = 2.5) and PC_71_BM were received from 1‐Material (Canada) and Nano‐C (United States). Zinc acetate dehydrate (purity > 99%), which is a precursor for ZnO, was purchased from Sigma‐Aldrich (United States), while molybdenum oxide (MoO_3_) was supplied from Tasco (United States) and used without further purification. Blend solutions of PTB7‐Th and PC_71_BM (PTB7‐Th:PC_71_BM = 1:1.5 by weight) were prepared using chlorobenzene (CB) as a solvent and 1,8‐diiodoctane (DIO) as an additive (CB:DIO = 97:3 by volume) at a solid concentration of 30 mg mL^−1^. The blend solutions were subject to vigorous stirring at room temperature for 24 h. The ZnO precursor solutions were prepared by dissolving zinc acetate dehydrate (109.75 mg) in the mixture of 2‐methoxyethanol (0.94 mL) and ethanolamine (0.06 mL), followed by stirring at 60 °C for 3 h and at room temperature for 12 h. PEI solutions were prepared by diluting with deionized water to be 0.01 wt%, followed by stirring at room temperature for 12 h.

*Device Fabrication and Thin Films*: Prior to device fabrication, a photolithography/etching process was applied to pattern ITO‐coated glass substrates in order to make ITO electrodes for electron collection. The patterned ITO‐glass substrates were subject to wet‐cleaning process with acetone and isopropyl alcohol inside a sonication bath (5510EDTH, Branson Ultrasonics Corp.). A nitrogen blow was used to dry the cleaned ITO‐glass substrates, followed by dry cleaning process using a UV‐ozone system (AH‐1700, Ahtech LTS) in order to remove any organic residues on the surface of the ITO‐glass substrates. Then the ZnO precursor solutions were spun on the cleaned ITO‐glass substrates and baked at 200 °C for 1 h in air ambient condition (typical laboratory condition), leading to the 30 nm thick ZnO layers. Next, the PTB7‐Th:PC_71_BM BHJ layers were spin coated on the ZnO layers inside a nitrogen‐filled glove box, followed by drying in the same glove box. After moving all samples to a vacuum chamber, MoO_3_ (10 nm) and Ag (80 nm) layers were thermally evaporated on the BHJ layers. The active area of devices was 0.09 or 0.055 cm^2^. The UCF (Hoya Corp.) was mounted on the glass part of devices by employing a high‐accuracy attachment control vision system in order to avoid any optical interference effect. All samples for TEM and XPS analysis were prepared in the same way as for the device fabrication.

*Device Measurement*: The optical absorption spectra of UCF and films were measured using an UV–vis spectrometer (Optizen 2120+, Mecasys Co., Ltd), while the surface of films were optically examined using an optical microscope (SV‐55, SOMETECH). The film thickness was measured using a surface profiler (Alpha Step 20, Tencor Instruments). The nanomorphology of the BHJ films was measured using a high resolution transmission electron microscope (FE‐TEM, Titan G2 ChemiSTEM Cs Probe, FEI Company), while a scanning transmission electron microscope (STEM) was used for the measurement of atom compositions in the BHJ films. The core‐level atom environments were measured with an X‐ray photoelectron spectrometer (XPS, ESCALAB 250Xi, Thermo Scientific). The current density–voltage (*J–V*) curves of solar cells were measured using a solar cell measurement system equipped with a solar simulator (class‐A, 92250A‐1000, Newport‐Oriel) and an electrometer (Keithley 2400). The intensity of simulated solar light was adjusted with a calibrated standard solar cell (BS‐520, Bunkoukeiki Co., Ltd) accredited by the Advanced Institute of Science and Technology (AIST, Japan), while the performance of solar cells measured in this work was subject to confirmation with a certified device by the Korea Institute of Energy Research (KIER, Korea).

## Supporting information

As a service to our authors and readers, this journal provides supporting information supplied by the authors. Such materials are peer reviewed and may be re‐organized for online delivery, but are not copy‐edited or typeset. Technical support issues arising from supporting information (other than missing files) should be addressed to the authors.

Supplementary
