# Supplementary material for: Significant Stability Enhancement in High‐Efficiency Polymer:Fullerene Bulk Heterojunction Solar Cells by Blocking Ultraviolet Photons from Solar Light
Source: Adv Sci (Weinh). 2015 Nov 17;3(4):1500269. doi: 10.1002/advs.201500269 (PMC5063255; doi:10.1002/advs.201500269)
Supplement: Supplementary file 1 — Supplementary [file ADVS-3-0h-s001.pdf]

Copyright WILEY-VCH Verlag GmbH & Co. KGaA, 69469 Weinheim, Germany, 2015.

ADVANCED SCIENCE
Open Access

Supporting Information

for *Adv. Sci.*, DOI: 10.1002/advs.201500269

Significant Stability Enhancement in High-Efficiency Polymer:Fullerene Bulk Heterojunction Solar Cells by Blocking Ultraviolet Photons from Solar Light

Jaehoon Jeong, Jooyeok Seo, Sungho Nam, Hyemi Han, Hwajeong Kim, Thomas D. Anthopoulos, Donal D. C. Bradley, and Youngkyoo Kim**

((Supporting Information can be included here using this template))

Copyright WILEY-VCH Verlag GmbH & Co. KGaA, 69469 Weinheim, Germany, 2013.

Supporting Information

Significant stability enhancement in high efficiency polymer:fullerene bulk heterojunction solar cells by blocking ultraviolet photons from solar light

Jaehoon Jeong,[a,#] *Jooyeok Seo,*[a,#] *Sungho Nam,*[a,b,#] *Hyemi Han,*[a] *Hwajeong Kim,*[a,c*] *Thomas D. Anthopoulos,*[b] *Donal D. C. Bradley,*[b] *and Youngkyoo Kim*[a,]*

[a] Organic Nanoelectronics Laboratory, Department of Chemical Engineering, Kyungpook National University, Daegu 702-701, Republic of Korea
[b] Center for Plastic Electronics, Department of Physics, Blackett Laboratory, Imperial College London, London SW7 2AZ, United Kingdom
[c] Priority Research Center, Research Institute of Advanced Energy Technology, Kyungpook National University, Daegu 702-701, Republic of Korea

*E-mail: ykimm@knu.ac.kr

[#]Equal Contribution: J.J., J.S., and S.N. contributed equally to this work.

Table S1. Summary of solar cell performances for glass/ITO/ZnO/PTB7-Th:$PC_{71}BM/MoO_3$/Ag solar cells without (a) and with (b) UV-cut filter (UCF).

	w/o UCF (under 100 mW/cm^2)			w/ UCF (under 100 mW/cm^2)			w/o UCF (under 80 mW/cm^2)		
	0 min	60 min	120 min	0 min	60 min	120 min	0 min	60 min	120 min
J_{SC} (mA/cm^2)	17.29	14.39	11.89	14.85	14.69	14.41	14.01	13.07	12.84
V_{OC} (V)	0.78	0.66	0.61	0.77	0.75	0.75	0.76	0.71	0.70
FF (%)	65.7	45.4	42.2	64.0	61.5	60.4	69.0	56.5	53.5
PCE (%)	8.87	4.31	3.06	7.33	6.77	6.53	7.35	5.25	4.81
R_S (Ω·cm^2)	80	150	180	90	90	90	80	120	130
R_{SH} (kΩ·cm^2)	12.9	2.2	2.7	6.8	8.3	6.6	28.4	4.5	9.4

Table S2. Summary of solar cell performances for glass/ITO/ZnO/PEI/PTB7-Th:$PC_{71}BM/MoO_3$/Ag solar cells without (a) and with (b) UV-cut filter (UCF).

	w/o UCF (under 100 mW/cm^2)				w/ UCF (under 100 mW/cm^2)					
	0 h	1 h	5 h	14 h	0 h	2 h	6 h	10 h	14 h	21 h
J_{SC} (mA/cm^2)	17.40	13.95	10.95	8.09	15.81	15.78	15.39	15.01	14.87	14.79
V_{OC} (V)	0.78	0.61	0.59	0.57	0.77	0.74	0.74	0.74	0.74	0.74
FF (%)	65.7	46.0	41.2	40.0	61.8	61.6	58.9	60.4	61.6	60.8
PCE (%)	8.91	3.91	2.66	1.84	7.53	7.19	6.71	6.71	6.78	6.65
R_S (Ω·cm^2)	80	150	210	370	150	120	140	110	100	100
R_{SH} (kΩ·cm^2)	5.7	2.2	1.8	2.6	8.4	4.2	11.0	5.4	5.5	7.3

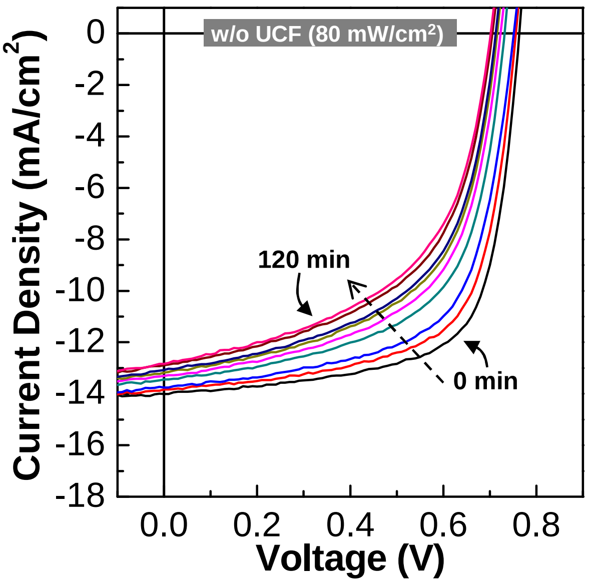

Figure S1. Current density – voltage (J-V) curves of glass/ITO/ZnO/PTB7-Th:$PC_{71}BM/MoO_3$/Ag solar cells under continuous illumination with simulated solar light (air mass 1.5G, 80 mW/cm^2) for 120 min without UCF.

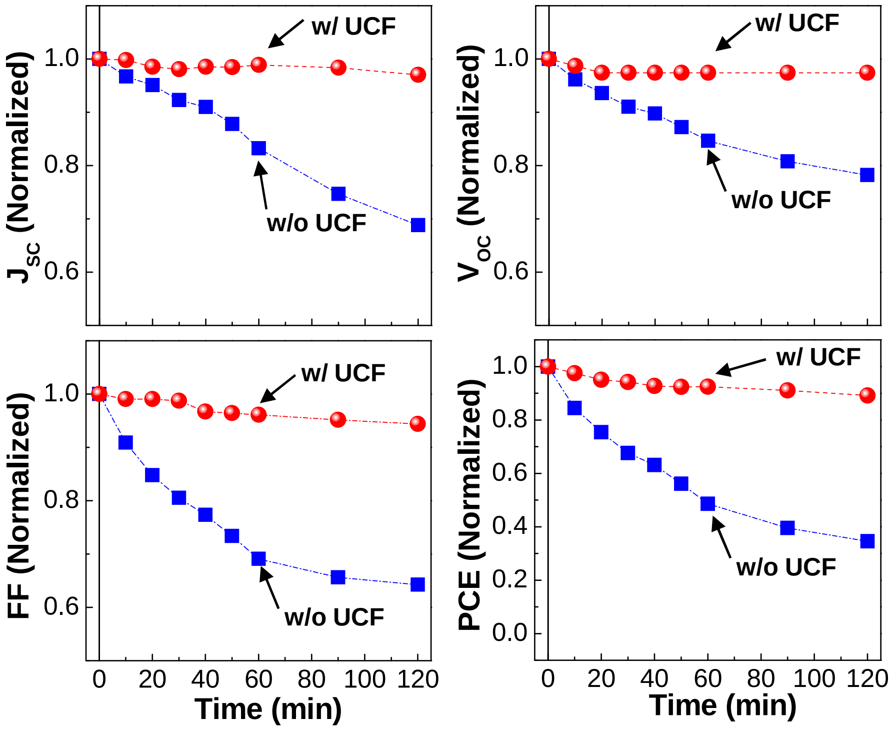

Figure S2. Normalized J_{SC}, V_{OC}, FF, PCE, R_S, and R_{SH} as a function of exposure time under simulated solar light (air mass 1.5G, 100 mW/cm^2) for glass/ITO/ZnO/PTB7-Th:$PC_{71}BM/MoO_3$/Ag solar cells with and without UCF (see original values in Figure 3).

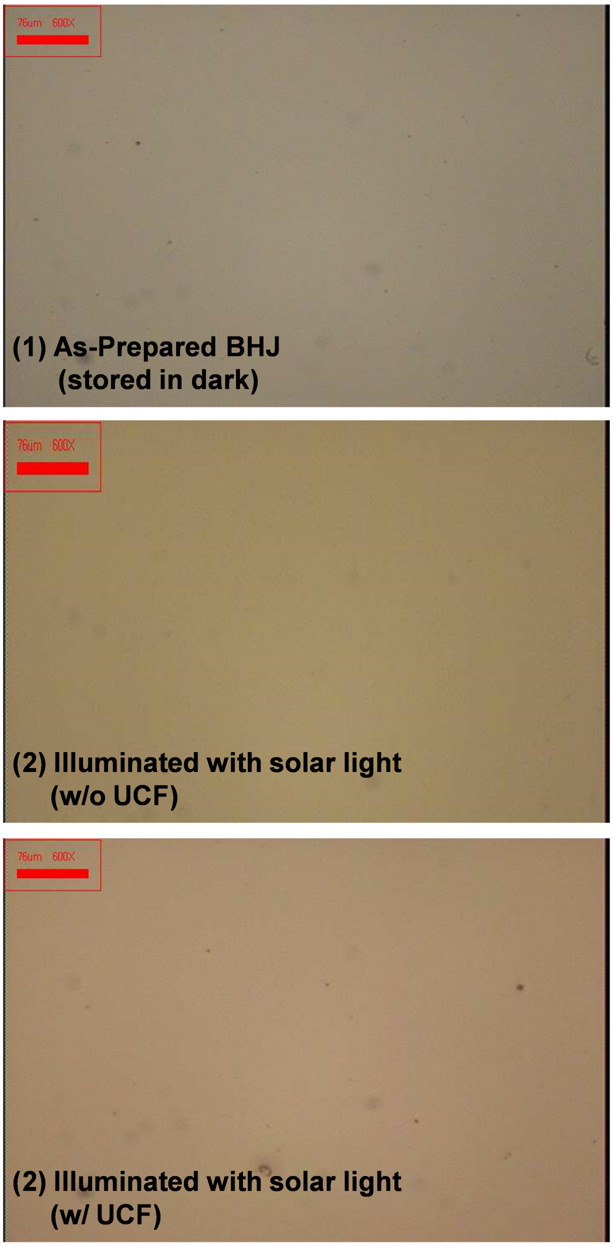

Figure S3. Optical microscope images for PTB7-Th:$PC_{71}BM$ BHJ films: (1) As-prepared, (2) illuminated with simulated solar light (air mass 1.5G, 100 mW/cm^2) without UCF for 120 min, and (3) illuminated with simulated solar light (air mass 1.5G, 100 mW/cm^2) with UCF for 120 min. The size of scale bar in red is 76 μm. Note that some big spots here come from defects in lenses of optical microscope.

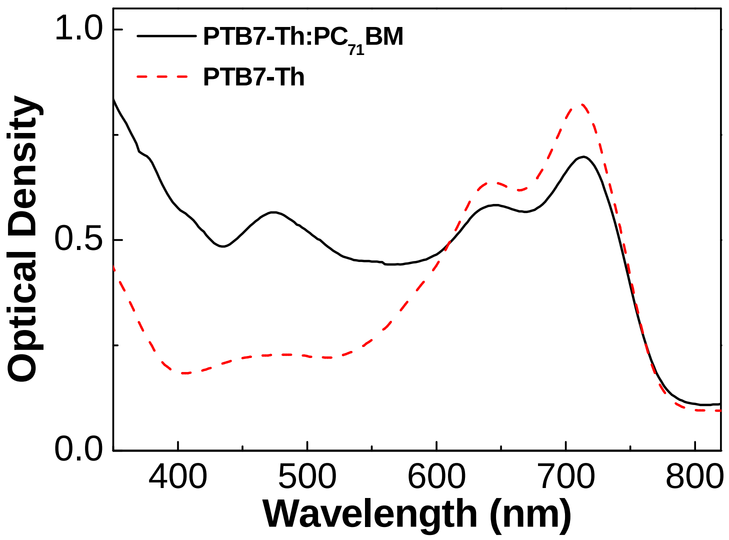

Figure S4. Optical absorption spectra of the pristine PTB7-Th film (red dashed line) and the PTB7-Th:$PC_{71}BM$ BHJ film (black solid line).

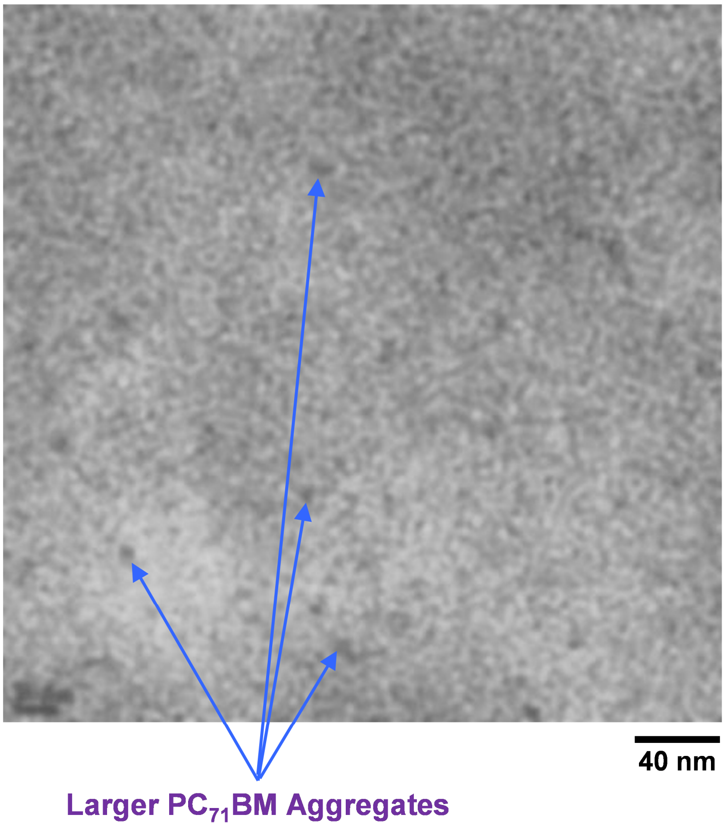

Figure S5. Enlarged TEM image (black & white) for the PTB7-Th:$PC_{71}BM$ BHJ film illuminated with simulated solar light (air mass 1.5G, 100 mW/cm^2) without UCF for 120 min. The blue arrows point the large aggregates of $PC_{71}BM$, which are considered to be formed by oxidative degradation of both components (preferably PTB7-Th) in the BHJ film.

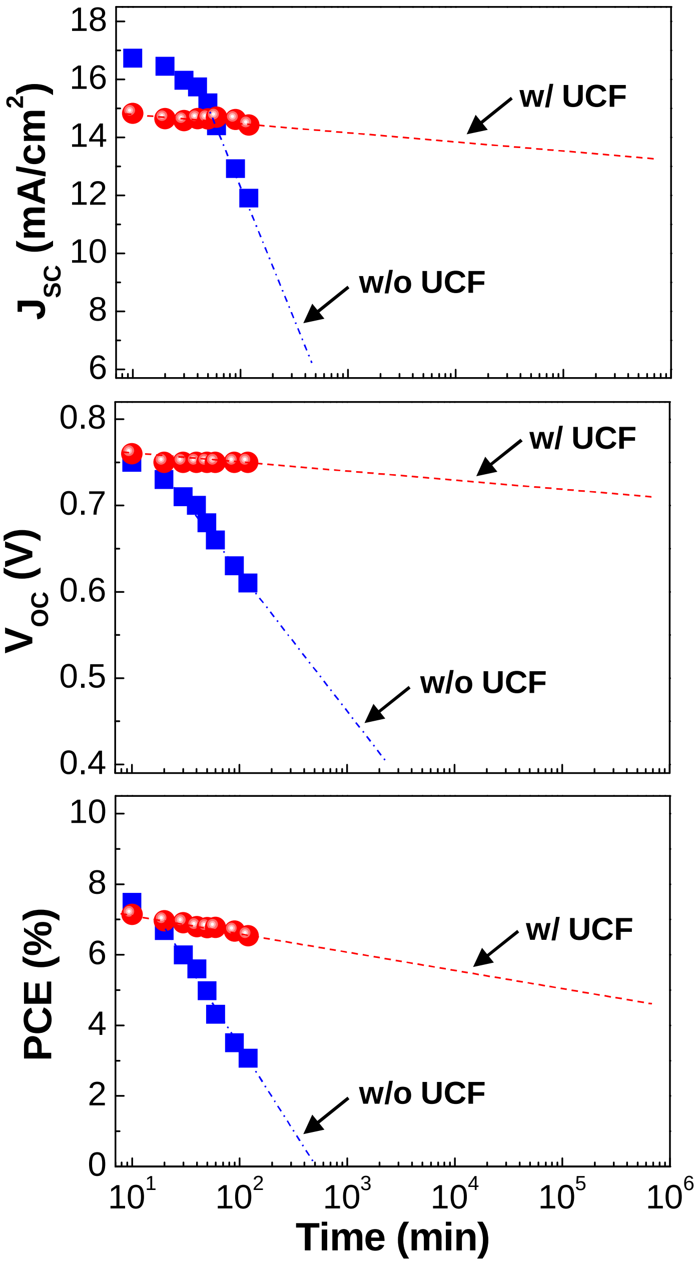

Figure S6. Projected J_{SC}, V_{OC}, and PCE as a function of exposure time under simulated solar light (air mass 1.5G, 100 mW/cm^2) for glass/ITO/ZnO/PTB7-Th:$PC_{71}BM/MoO_3$/Ag solar cells with and without UCF.

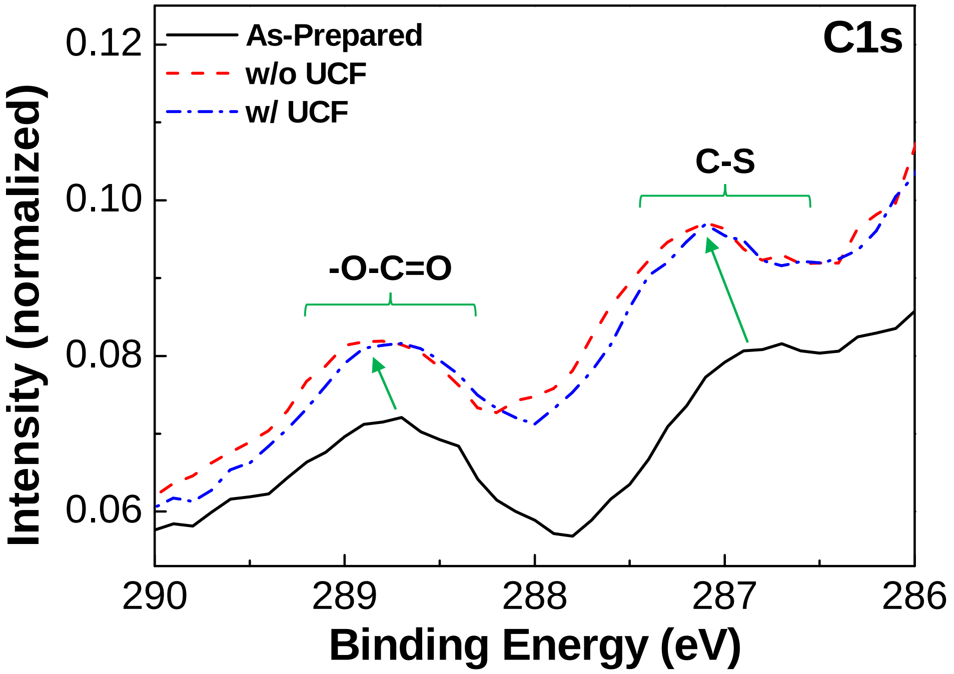

Figure S7. Enlarged C1s XPS spectra for –O-C=O and C-S groups in the PTB7-Th:$PC_{71}BM$ BHJ films: (1) As-prepared (black solid line), (2) illuminated with simulated solar light (air mass 1.5G, 100 mW/cm^2) without UCF for 120 min (red dashed line), and (3) illuminated with simulated solar light (air mass 1.5G, 100 mW/cm^2) with UCF for 120 min (blue dash-dot line). Green arrows denote the shift of C1s peaks in the as-coated film (1) after illumination, indicating the environmental change of carbon atoms in the –O-C=O and C-S groups of the BHJ films after illumination.

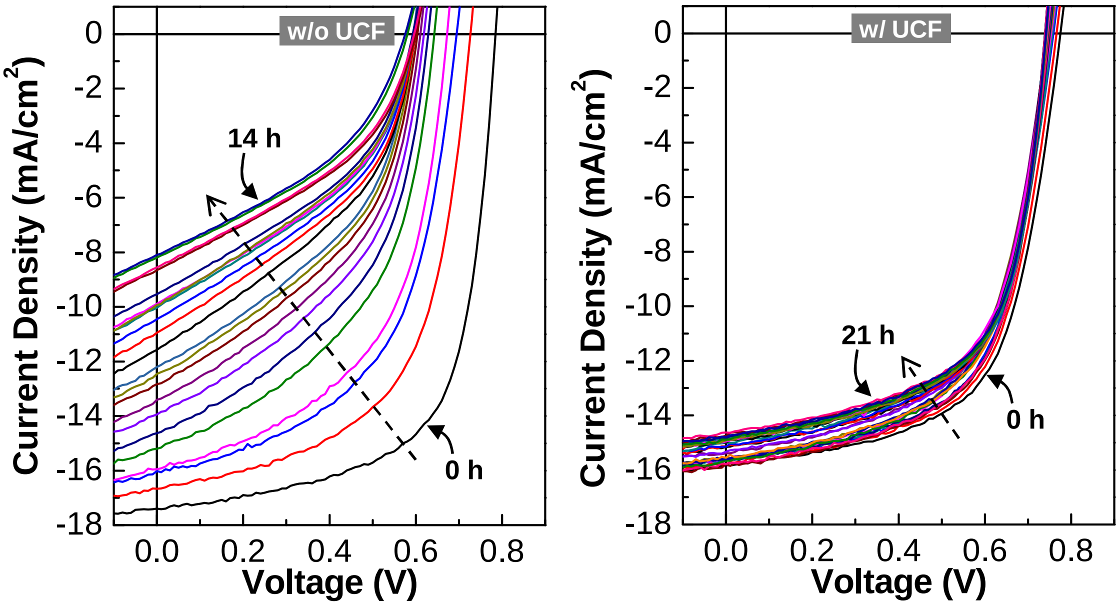

Figure S8. Current density – voltage (J-V) curves of glass/ITO/ZnO/PTB7-Th:$PC_{71}BM$/MoO_3/Ag solar cells under continuous illumination with simulated solar light (air mass 1.5G, 100 mW/cm^2): (left) without UCF for 14 h and (right) with UCF for 21 h. Note that the sorted J-V curves appear in Figure 7.
